# 

**Published:** 1862-07

**Authors:** 


### CONTENTS.

ORIGINAL ESSAYS AND COMMUNICATIONS.

Our Duty to our Patients, the People at Large, and to our Profession. By Dr. W. H. Atkinson .......... 293
Destroying Nerves and filling Cavities. By W. H. Shadoan .......... 298
Who first suggested Headed Pins for Artificial Teeth? .......... 301
Care of the Teeth. By J. Taft .......... 304

SELECTIONS.

Common Colds .......... 306
Swaging and Finishing Plates. By Ambler Tees .......... 307
Utility of Coffee in Soldiers' Diet .......... 310
Becquerel's Galvanic Apparatus .......... 310
Dentition and its Derangements. By J. Jacobi, M. D. .......... 312
Creosote Collodion .......... 319
Arnica Hair Wash .......... 320
Poisoning by Chloroform .......... 320
Solidified Creosote .......... 321
Some Uses of Paraffin for Chemical Purposes .......... 322
India Rubber .......... 323
Practical Hints. By J. D White .......... 324
McLean's Neuralgic Liniment .......... 325
On Quackery. By W. H. Matchtt, M. D., Ithaca, O .......... 326

EDITORIAL.

Careless Writing .......... 336
Questions of a Correspondent .......... 338
A Little Experience with Leeches .......... 339
Templars' Magazine .......... 340

